# The Immunomodulatory Effect of Alpha-Lipoic Acid in Autoimmune Diseases

**DOI:** 10.1155/2019/8086257

**Published:** 2019-03-20

**Authors:** Wei Liu, Lian-jie Shi, Sheng-guang Li

**Affiliations:** ^1^Department of Respiratory and Critical Care Medicine, The 900th Hospital of the Joint Logistic Support Force, PLA, Fujian Medical University, Fuzhou 350025, China; ^2^Department of Rheumatology and immunology, Peking University International Hospital, Beijing 102206, China

## Abstract

Αlpha-lipoic acid is a naturally occurring antioxidant in human body and has been widely used as an antioxidant clinically. Accumulating evidences suggested that *α*-lipoic acid might have immunomodulatory effects on both adaptive and innate immune systems. This review focuses on the evidences and potential targets involved in the immunomodulatory effects of *α*-lipoic acid. It highlights the fact that *α*-lipoic acid may have beneficial effects in autoimmune diseases once the immunomodulatory effects can be confirmed by further investigation.

## 1. Background

Αlpha-lipoic acid (ALA) is a naturally occurring dithiol compound that is wildly synthesized in the mitochondrion by plants and animals. Physiologically, ALA is a cofactor for *α*-ketoglutarate dehydrogenase complex to protect mitochondria from oxidative attack.

ALA and dihydrolipoic acid (DHLA) are the oxidized form and reduced form of LA, respectively. They are a pair of powerful redox couple which can directly scavenge reactive oxygen species (ROS), chelate metals, and regenerate other antioxidants to show antioxidant biochemical properties. With both liposoluble and water-soluble dual properties, ALA and DHLA can fully function intracellularly and extracellularly [1, 2].

Based on its cogent antioxidant properties and proven safety, ALA has been widely used to treat oxidative stress associated diseases, such as diabetes, neurological diseases, and cardiovascular diseases. More and more studies on LA had been performed; better understanding had been achieved towards the mechanisms of molecules, including the fact that ALA stimulated glucose uptake in insulin-sensitive cells and enhanced both the antioxidant defenses and the function of endothelial vascular cells [3]. Several lines of evidence suggested that ALA might have immunomodulatory effect.

With the progress of investigation of basic and clinical immunology, the role of oxidative stress on the pathogenesis of some autoimmune diseases has been generally recognized and interaction of ROS with the immune system well proven. On one hand, ROS may have a physiological role in signal transduction of all kinds of immune cells. For example, macrophage produced ROS to kill bacteria and regulatory T cells (Treg) released ROS to suppress activation of other T cells [4]. On the other hand, in pathological status, immune cells produce excessive ROS which exacerbated inflammation and broke balance in the immune system. For example, oxidative stress was one of the contributors to immune system dysregulation and dysfunction [5], which in turn led to deteriorate oxidative stress in systemic lupus erythematosus (SLE) [6, 7]. Both oxidative stress and immune dysfunction participated in the development and progression of SLE. Recently, redox-controlled activation of the mechanistic target of rapamycin (mTOR) has been recognized to play a critical regulatory role in the immune system [8], which highly implies that mTOR is a key bridge of metabolic stress and autoimmunity. It can be speculated that the antioxidant may be used for the treatment of certain autoimmune diseases based on evidence that ROS clearance played a role in immune regulation. Here in, we summarize the evidence of the immunomodulatory effects of ALA and possible mechanisms involved by literature review.

### 1.1. The Involved Signaling Pathways of ALA

#### 1.1.1. IKK*β*, Ras/Erk1/2, and PI3K/Akt/mTOR Signaling Pathways

The mTOR, which could drive expansion of plasmablast [9] and T follicular helper (Tfh) cells [10], induce the differentiation of Th1 and Th17 [11], and restrict the differentiation of Treg [12] and CD8 memory T cells [13], is an essential mediator of immunity.

ALA had been shown to regulate upstream kinases of mTOR in multiple pathological conditions [1].

ALA blocked TNF-*α* induced IKK/NF-*κ*B signaling cascades in RA-FLS and human umbilical vein endothelial cells (HUVECs) [14, 15]. TNF-*α* activated mTORC1 pathway via IKK*β* activation in tumor angiogenesis and insulin resistance [16, 17]. Therefore, it can be speculated that ALA may suppress IKK*β*-mediated activation of mTORC1.

ALA inhibited Erk signaling to improve atherosclerotic lesions and inhibit vascular smooth muscle cells proliferation [18]. ALA also inhibited activation of Erk mediated by 5-hydroxytryptamine (5-HT) [19] and epidermal growth factor (EGF), basic fibroblast growth factor (bFGF), and platelet-derived growth factor (PDGF) [20]. The activation of Akt/S6K1 and Erk suppressed by ALA attenuated hepatic stellate cell activation and ROS generation stimulated by TGF-*β*/PDGF [21].

However, Erk signaling activated by ALA has been reported to protect cardiovascular system and nervous system. ALA increased heme oxygenase-1 (HO-1) to protect vascular smooth muscle cells [22], ameliorated glucose/glucose oxidase- (G/GO-) induced injury of rat cardiomyoblast [23], inhibited adipocyte differentiation [24], protected cortical neurons from 4-hydroxy-2-nonenal- (HNE-) mediated oxidative damage and neurotoxicity [25], and promoted neurite outgrowth via activation of Erk [26].

The effect of bidirectional regulation of Erk1/2 kinases on mouse fibroblasts mediated by ALA was dependent on the cell culture medium containing serum or not [27, 28], which, to some extent, can interpret the fact that ALA regulated the same kinase in different directions in different pathological states.

ALA activated Akt kinase to protect pancreatic beta cells from hydrogen peroxide-mediated oxidative stress [29]. In rat L6 muscle cells, ALA mitigated insulin resistance via Akt activation and Erk inhibition [30].

ALA enhanced apoptosis and suppressed cell proliferation of human breast cancer cell line [31]. ALA also induced hepatoma cells apoptosis [32] to exert antitumor effects by suppression of PI3K/Akt pathway. ALA also inhibited leptin production of adipocytes and ameliorated insulin resistance of Goto-Kakizaki (GK) rat to improve disorders of glucose and lipid metabolism [33, 34].

Cytoprotective effect of ALA also could be mediated through phosphorylation of Akt kinase to ameliorate endoplasmic reticulum stress-induced FRTL5 thyroid cell death [35], protect neurons from injury induced by bupivacaine, amyloid and hydrogen peroxide [36, 37], reduce ischemia-reperfusion injury [38, 39] and oxidative stress injury of rat L6 muscle cells induced by TNF*α* and palmitate [30], decrease hydrogen peroxide-induced apoptosis of pancreatic beta cells [29], attenuate LPS-induced cardiac dysfunction [40], monocyte activation, and acute inflammatory responses [41], and ameliorate vascular endothelial dysfunction [42, 43].

#### 1.1.2. AMPK Signaling Pathway

ALA has been reported to activate AMPK to upregulate adipose triglyceride lipase (ATGL) to reduce body weight and visceral fat content of diabetic mice [44].

Through AMPK/mTORC1/S6K1 signaling pathway, leucine and glucose induced insulin resistance which could be attenuated by ALA via TSC2-mTOR inhibitors-phosphorylation [45, 46] and AMPK activation [47] in skeletal muscle. ALA also activated AMPK to downregulate expression of S6K1 [48] leading to inhibition of insulin secretion in pancreatic beta cells, which implies involvement of mTOR.

However, ALA was also reported to inhibit the phosphorylation of AMPK, which suppressed appetite and prevented obesity in the hypothalamus [49–51] consistent with the effect of ALA on peripheral tissue to improve insulin resistance and decrease lipid accumulation and lipogenesis.

Overall, ALA regulated some upstream kinase of mTOR in inconsistent directions in diverse cell types of different diseases. It has been proven that mTOR can modulate T cell differentiation and inhibit Treg cells which are deficient in SLE patients [52, 53]. N-acetylcysteine (NAC), a well-known antioxidant, has been reported to inhibit mTOR in vitro [54] and improve the outcome of murine lupus [55] and even SLE patients [56]. It has also been observed that disease activity could be reduced and Treg populations could be reversed by mTOR blockade in treating SLE patients [57]. Although these existing indirect evidence was tempting to conclude that ALA had effect on regulation of mTOR signaling, regulation of the mTOR pathway by ALA in immune cells is worthy of further investigation for patients with autoimmune diseases of high relapse rate and poor responsiveness to traditional treatment.

## 2. Effects of ALA on Immune System

**Figure 1:**
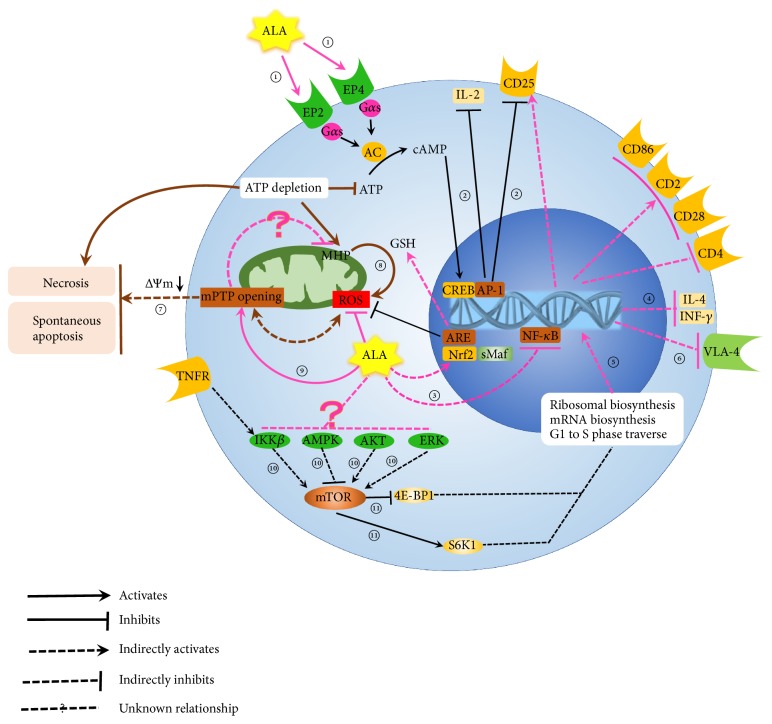
*Effect of ALA on T cell*. ① ALA increase cAMP synthesis through activation of prostaglandin receptors (EP2 and EP4) in peripheral blood T cells. ② The expression of IL-2 and IL-2R*α* (CD25) could be inhibited when the level of cAMP was increased by ALA. ③ ALA could inhibit NF-*κ*B activation induced by TNF in Jurkat T cells. ④ ALA could suppress production of interferon-*γ* (IFN-*γ*) and interleukin -4 (IL-4). ⑤ ALA could induce lymphocyte progression from G0/G1 to S phase. ⑥ ALA was also reported to reduce migration of T cell, lymphocyte and monocyte of models of MS, and Jurkat T cells, which were associated with down-regulated expression of very late activation-4 antigen (VLA-4). ⑦ Uncontrolled mPTP opening leads to decrease ∆Ψm irreversibly until dissipation which results in apoptosis and necrosis of cells. ⑧ Persistent MHP in SLE T cells could enhance ROS production. ⑨ Studies showed that ALA and DHLA promoted mPTP opening in mitochondria of rat liver. ⑩ mTORC1 was a central common regulator of a complex signaling network In cytoplasm, in which Ras/Erk, PI3K/Akt, and IKK*β* activated mTORC1 while Dsh/GSK3 and LKBl/AMPK inactivated mTORC1. ⑪ Activated mTORC promoted protein synthesis by phosphorylating the eukaryotic initiation factor 4E-binding protein 1 (4E-BP1) and p70 ribosomal S6 kinase 1 (S6K1).

The proven effects of ALA on adaptive immune cells, including T and B cells, are briefly summarized in Table 1 and Figure 1, and the proven effects on innate immune cells including NK cells, macrophages, and monocytes are summarized in Table 1, all of which will be discussed in detail below.

### 2.1. Effects on Adaptive Immune Cells

#### 2.1.1. Effects on T Cells

Multiple sclerosis (MS) is an autoimmune disease in central nervous system, which is characterized by the migration and the long-term survival of myelin-specific T lymphocytes into the central nervous system (CNS). A common model of MS is experimental autoimmune encephalomyelitis (EAE). Studies demonstrated the beneficial effects of ALA on treating EAE by suppressing the infiltration of inflammatory cells [58–61]. Recently, Wang and colleagues [60] reported that ALA reduced the number of Th17 and Th1 cells in CNS and increased the number of splenic Treg cells in EAE mice. It highly implied that ALA showed immunomodulatory effects on differentiation and proliferation of T cells. ALA has also been reported to increase cAMP synthesis through activation of prostaglandin receptors (EP2 and EP4) in peripheral blood T cells [62] (Figure 1, ①). The elevated levels of intracellular cAMP decreased the expression of IL-2 and IL-2R*α* (CD25) [63] (Figure 1, ②), which in turn affected proliferation and activation of T cells [64].

More studies indicated that ALA could regulate function of T cells in many ways. ALA could ameliorate the impaired mitochondrial function of CD4^+^T cells of acquired immunodeficiency syndrome (AIDS) patients [65], downregulate the expression of CD4 molecules of human peripheral blood T cells [66], inhibit nuclear factor kappa B (NF-*κ*B) activation induced by tumor necrosis factor-alpha (TNF) in Jurkat T cells [67] (Figure 1, ③), suppress production of interferon-*γ* (IFN-*γ*) and interleukin-4 (IL-4) (Figure 1, ④) by CD4^+^T cells to reduce the severity of atopic dermatitis lesions in mice model [68], and induce lymphocyte progression from G0/G1 to S phase (Figure 1, ⑤), which might be related to restore the function of immune system in advanced cancer patients [69].

Besides effects on T cell proliferation, differentiation, and the cytokines produced by them, ALA could also inhibit migration of T cells. Ying and colleagues found that ALA could directly reduce T cell migration in response to chemokines to reduce T cell numbers in atherosclerotic plaque in models of established atherosclerosis [70]. ALA was also reported to reduce migration of T cell [58, 71], lymphocyte and monocyte of models of MS [61], and Jurkat T cells [72], which were associated with downregulated expression of very late activation-4 antigen (VLA-4) (Figure 1, ⑥) and inhibition of MMP-9 activity by ALA [72].

#### 2.1.2. Effects on B Cells

The studies showed that ALA supplement might play a role in high fat diet mice to prevent the development of oxidative stress and to attenuate B-cell injury by increasing the gene expression of the B-cell receptor (BCR) signaling pathway and decrease the apoptotic percentage of splenic B lymphocytes [73], which was also relevant to the improvement of gene expression level of BCR [74]. It has been proven that ALA increased the number of splenic B cells in endotoxemia mice [75] and reduced total serum IgE levels of atopic dermatitis mice model [68]. These experiments suggest that ALA plays a regulating role on proliferation, apoptosis and function of B cells (Table 1).

#### 2.1.3. Effects on Innate Immune Cells

*Natural Killer Cell (NK Cell), Macrophage, and Monocyte (See Table 2)*. Cytotoxicity and cytokines secretion are two main functions of NK cells. The former is associated with the release of granzymes (perforin and proteases) from their cytoplasm. INF-*γ* secretion is a representative of the latter. INF-*γ* is a potent macrophage activator for both phagocytosis and lysis. IFN-*γ* secretion induced by IL-12/IL-18 and cellular cytotoxicity in NK cells could be inhibited by ALA, which increased cAMP production via G protein-coupled receptors- (GPCRs-) dependent and GPCRs-independent mechanisms [62, 76, 77]. In addition, it has also been demonstrated that both cAMP and cAMP-inducing agents (PGE1, theophylline, and histamine) suppressed cytolytic function of NK cells [78]. PGE2, another cAMP elevating agent, also suppressed cytotoxicity and IFN-*γ* production induced by IL-15 [79]. Therefore, ALA could suppress NK function in a few ways.

It has also been found that ALA regulated activation, phagocytosis, and migration of macrophage by either direct or indirect means. ALA inhibited the phagocytosis of myelin by macrophages [80], which was the main autoantigen in EAE mice, and decreased the production of monocyte chemotactic protein 1 (MCP-1) and TNF*α* induced by lipopolysaccharide (LPS) of macrophages [81, 82]. Also, ALA decreased monocytes infiltration into the CNS and stabilized brain endothelial cells in EAE rat [61], which might be associated with the downregulated intracellular adhesion molecule-1 (ICAM-1) expression of monocytes [83] and the upregulated vascular cell adhesion molecule-1 (VCAM-1) expression of endothelial cells [84]. In addition, ALA could induce the expression of heme oxygenase-1(HO-1) by nuclear factor-erythroid 2-related factor 2 (Nrf2) in human monocytic cells [85].

## 3. Other Potential Targets of ALA Immunomodulatory Effects

ALA has been widely used for decades in clinic and studied in various experimental models. Therefore, we found the potential targets for immunomodulatory effects of ALA in these researches.

### 3.1. Mitochondrial Membrane Potential (∆Ψm)

Mitochondria provide place for the citric acid cycle and oxidative phosphorylation, which is the energy station of cells and is involved in cell differentiation, cell cycle regulation, and cell death. The stability of ∆Ψm is essential for the maintenance of normal physiological function of cells. The electron transport chain and the F_0_F_1_-ATPase complex maintain an electrochemical gradient namely “∆Ψm”, and vice versa, ∆Ψm tightly regulates the production of ROS and ATP synthesis [86]. Mitochondrial permeability transition pore (mPTP) is a series of protein channels which are located in the inner and outer mitochondrial membrane. mPTP closes completely to stabilize ∆Ψm whereas mPTP opens transiently to a low conductance state to result in lowering ∆Ψm. Uncontrollable mPTP opening leads to decrease ∆Ψm irreversibly until dissipation which results in apoptosis and necrosis of cells [87, 88] (Figure 1, ⑦). In the process of both activation and apoptosis of T lymphocytes, ∆Ψm was transiently reversibly elevated, that is what mitochondrial hyperpolarization (MHP) in physiological status should be [89]. However, persistent MHP in T cells of SLE patients would enhance ROS production (Figure 1 ⑧), which resulted in activation of macrophages [90] and dendritic cells [91] to exacerbate inflammation [92, 93] and resulted in ATP depletion which increased IL-10 production and spontaneous apoptosis of T cells [94]. T cell apoptosis not only provided a source of nuclear antigens but also was correlated with SLE disease activity [95, 96]. Increased production of IL-10 could promote T cell apoptosis [97] and contribute to the production of autoantibodies by hyperactive SLE B lymphocytes [98, 99]. There were proofs showing that ALA and DHLA promoted mPTP opening of mitochondria in rat liver [3, 93, 100] (Figure 1, ⑨). Thus, the authors speculate that ALA may attenuate mitochondrial dysfunction in SLE from several aspects. ALA opens mPTP to reduce ΔΨm, which improves pathological MHP of SLE T cells and directly quenches ROS to correct dysfunction of T cells and B cells.

### 3.2. Neutrophil Extracellular Traps (NETs)

Neutrophils play a very important role in innate immune system and are the first leukocytes to be recruited to the site of infection to eliminate pathogens by multiple mechanisms which include phagocytosis, inflammatory mediators secretion, and NETs release which is also known as NETosis. NETs are composed of nucleic acids, histone proteins, and granule proteins with or without death of neutrophils [101]. NETs only fight against pathogens but also have been implicated in pathogenesis of the autoimmune diseases (e.g., SLE [102, 103], RA [104, 105], psoriasis [106], and autoimmune small-vessel vasculitis [107]) and thrombosis [108]. Nuclear material of NETs components became autoantigens after it was extruded from the cell to induce autoantibodies production [109]. NETs formation was dependent on autophagy and ROS generation [110, 111] and regulated by mTOR signaling pathway [112, 113]. It has been speculated that ALA may be capable of quenching ROS and regulating mTOR signaling, which suggests that it may have beneficial effects on NETs formation to reduce the autoantibodies production and protect vascular endothelium.

### 3.3. Nrf2 Signaling Pathway

ALA is well recognized to be an activator of Nrf2 signaling [1, 114]. The nuclear factor-erythroid 2-related factor 2 (Nrf2), a central regulator of cellular resistance to oxidant stress, binds antioxidant response element (ARE) to regulate expression of a lot of ARE-containing genes to play a pivotal role in control of oxidant homeostasis [115]. There is a little evidence to show association between Nrf2 signaling and pathogenesis of autoimmune disease. It has been demonstrated that Nrf2-deficient female mice developed severe nephritis similar to lupus [116] and Nrf2 gene variant was relevant with nephritis in childhood-onset SLE patients [117]. Nrf2 (-/-) mice developed regenerative immune-mediated hemolytic anemia [118] and disruption of Nrf2 aggravated [119] while activation of Nrf2 attenuated [120] neuroinflammatory disorders in EAE. Hence, ALA may have regulatory effects in immune system via Nrf2 pathway.

## 4. Safety of ALA

ALA, a naturally occurring antioxidant in human body and available from common dietary sources, has been used to treat diabetic neuropathy and retinopathy for over 50 years in Germany. A number of clinical trials have reported that oral LA supplementation up to 2400 mg/d and intravenous LA supplementation up to 600 mg/d for three weeks showing no adverse effects versus placebo [1, 2]. Moreover, flexible regulatory effects of ALA have been shown by Sen and colleagues that ALA promoted apoptosis induced by Fas in Jurkat cells but not healthy peripheral blood lymphocytes [121]. The data of these studies supports the safety of ALA.

## 5. Summary

In conclusion, ALA, a natural ingredient of human body, not only acts as a powerful antioxidant but also is able to regulate the immune system in either direct or indirect ways. Studies reviewed above might suggest that ALA is used to treat autoimmune diseases including SLE, RA, and primary vasculitis as well as MS. The current therapies for systemic rheumatic diseases are effective. However, there was still a high percent of patients with not enough or no response to the therapies. Therefore, if the immunomodulatory effects of ALA could be confirmed by further investigation, it might have beneficial effects in conjunction with the current treatment of rheumatic diseases.

## Figures and Tables

**Table 1:** Evidence of ALA on adaptive immune cells.

		T cell	B cell
Animal model	EAE	Decrease the number of Th17 and Th1 in CNS; Increase Treg numbers in spleen; Reduce migration.	
	High fat diet mice	Recover transcriptional levels of the differentiation-related genes of jejunal T cells.	Restore transcriptional levels of BCR signaling pathway relating genes; Decrease the apoptotic percentage of splenic B lymphocytes.
	Atopic dermatitis	Suppress production of IFN-*γ* and IL-4 by CD4+T.	Reduce total serum IgE levels.
	Models of established atherosclerosis	Reduce T cell migration in response to chemokines.	
	Endotoxemia mice		Increase the number of splenic B cells.

Patients	AIDS	Increase the number of Th cells; Improve the lymphocyte proliferation response; Ameliorate the impaired mitochondrial function of CD4^+^T cells.	
	Advanced cancer	Induce lymphocyte progression from G0/G1 to S phase.	
	Jurkat T cells	Inhibit NF-*κ*B activation induced by TNF Reduce migration.	

Normal human		Increase cAMP which affects proliferation and activation of T cells; Down-regulate the expression of CD4 molecules; Reduce migration.	

ALA: *α*-lipoic acid.

EAE: experimental autoimmune encephalomyelitis.

Th17: T helper cell 17.

Th1: T helper cell 1.

CNS: central nervous system.

Treg: regulatory T cells.

BCR: B-cell receptor.

IFN-*γ*: interferon-*γ*.

AIDS: acquired immunodeficiency syndrome.

NF-*κ*B: nuclear factor kappa B.

TNF: tumor necrosis factor.

cAMP: cyclic adenosine monophosphate.

**Table 2:** Evidence of ALA on innate immune cells.

		NK cell	Macrophage	Monocyte
Animal model	EAE		Inhibit the phagocytosis of myelin.	Decrease monocytes infiltration into the CNS.
	High fat diet mice		Suppress infiltration and activation of macrophage to attenuate visceral adipose inflammation.	
	BMDM or RAW 264.7		Decrease the production of MCP-1 and TNF-*α* induced by LPS.	

Normal human		Increase cAMP production to suppress cytotoxicity and IFN-*γ* production.		Induce the expression of HO-1 by Nrf2.

ALA: *α*-lipoic acid.

NK cell: natural killer cell.

EAE: experimental autoimmune encephalomyelitis.

CNS: central nervous system.

BMDM: bone marrow-derived macrophages.

MCP-1: monocyte chemotactic protein 1.

TNF-*α*: tumor necrosis factor-alpha.

LPS: lipopolysaccharide.

cAMP: cyclic adenosine monophosphate.

IFN-*γ*: interferon-*γ*.

HO-1: heme oxygenase-1.

Nrf2: nuclear factor-erythroid 2-related factor.
